# Risk Factors for a High Comprehensive Complication Index after Splenectomy Plus Pericardial Devascularization for Portal Hypertension

**DOI:** 10.5152/tjg.2023.22756

**Published:** 2023-10-01

**Authors:** Yafei Zhang, Hongwei Lu, Hong Ji, Yiming Li

**Affiliations:** 1 Department of Hepatobiliary Surgery, The First Affiliated Hospital of Zhengzhou University, Zhengzhou, China; 2 Department of General Surgery, The Second Affiliated Hospital of Xi’an Jiaotong University, Xi’an, China

**Keywords:** Comprehensive complication index, splenectomy, pericardial devascularization, portal hypertension

## Abstract

**Background/Aims:**

Mathematical integration of all complications from the Clavien–Dindo classification into one number called the comprehensive complication index provides a novel method to capture morbidity. This objective of this study was to compare the evaluations of complications between the novel comprehensive complication index and Clavien–Dindo classification for portal hypertension patients who underwent splenectomy plus pericardial devascularization.

**Materials and Methods:**

Patients treated with either splenectomy plus simplified pericardial devascularization or splenectomy plus traditional pericardial devascularization were included retrospectively. Correlation and logistic regression analyses of the postoperative hospital stay and total hospitalization expense were compared between the comprehensive complication index and Clavien–Dindo classification. The cumulative sum-comprehensive complication index was generated and compared between operation types.

**Results:**

The Child–Pugh classification at admission, spleen thickness, and intraoperative blood loss were risk factors for high comprehensive complication index. Comprehensive complication index showed a stronger relationship with the postoperative hospital stay and total hospitalization expense than the Clavien–Dindo classification. Logistic regression analysis of the postoperative hospital stay demonstrated that the *R*^2^ values for the comprehensive complication index and Clavien–Dindo classification were 0.15 and 0.14, respectively. The cumulative sum-comprehensive complication index graph showed a steady dynamic decrease in the cumulative sum score for the individual operation type, with splenectomy plus simplified pericardial devascularization revealing a more notable decrease than splenectomy plus traditional pericardial devascularization.

**Conclusions:**

Comprehensive complication index is an excellent method to assess postoperative morbidity in portal hypertension patients. The cumulative sum-comprehensive complication index chart can better dynamically monitor and compare different operation types. Splenectomy plus simplified pericardial devascularization is better than splenectomy plus traditional pericardial devascularization at decreasing cumulative sum-comprehensive complication index.

**Main Points**

- The risk factors for a high comprehensive complication index (CCI) score were the Child–Pugh classification at admission, spleen thickness, and intraoperative blood loss.
- The CCI had a stronger correlation with the postoperative hospital stay and total hospitalization expense than the Clavien–Dindo classification (CDC).
- Postoperative hospital stay was greater than that of the CDC. All operation types showed a dynamic decrease in the cumulative sum (CUSUM) score in the CUSUM–CCI model, although the decrease obtained with simplified splenectomy plus pericardial devascularization (SPD) was more notable than that obtained with traditional SPD.

## INTRODUCTION

Portal hypertension (PH) is a major complication of cirrhosis that manifests as ascites, splenomegaly, hypersplenism, and esophageal varices. The incidence and mortality of severe esophagogastric variceal hemorrhage are extremely high and seriously threaten the life and health of patients.^1^ Splenectomy plus pericardial devascularization (SPD) is the main operative method for the prevention and treatment of portal hypertension. Although great progress has been made in surgical techniques and methods in recent years, the incidence of perioperative complications is still extremely high. Therefore, control and monitoring of complications are necessary.^2,3^ Unfortunately, few studies have comprehensively evaluated the postoperative complications of PH patients undergoing SPD.

To rank a complication in an objective and reproducible manner, the Clavien–Dindo classification (CDC) is conducted based on the therapy used to correct a specific complication and has been widely applied to classify complications in many fields of surgery.^4–7^ However, the CDC reports only the most severe complications and excludes other collateral complications, although typically severe complications are accompanied by various minor complications. Therefore, the CDC cannot reflect the comprehensive effect of the total complications on patients. In addition, the CDC is an ordinal and not a continuous scale; thus, the differences between 2 adjacent grades are not necessarily equal, which limits its application as an outcome variable in statistics.^4^

Recently, the comprehensive complication index (CCI) was developed and validated in a large cohort of patients who underwent abdominal surgery. This index has higher sensitivity than the traditional evaluation method for postoperative complications.^8–10^ The CCI is a novel method that mathematically integrates all complications graded by the conventional CDC into one number regardless of the number and severity of the complications to capture the overall burden of an operation. Another prominent aspect of the CCI is that it is a continuous linear scale ranging from 0 (no complications) to 100 (death), can easily quantify complications and can be included in multifactor analyses. Thus, the CCI is the most attractive method for evaluation of postoperative complications. The CCI has been applied in abdominal surgery and in the context of randomized controlled trials for patients undergoing esophagectomy and has achieved better results.^11^ The cumulative sum (CUSUM) control technique uses a graphical tool to detect data trends and monitor surgical performances. Because the CCI represents the overall magnitude of all complications, continuous detection of the CCI can mirror the surgical performance.^11,12^

The aim of this retrospective study was to compare the evaluations of complications between the novel CCI and the conventional CDC in a large cohort of SPD patients and to identify risk factors for a high postoperative CCI score (CCI at 1 month ≥26.2). Then, we applied the CUSUM-CCI chart to monitor and compare complications between different operation types.

## MATERIALS AND METHODS

### Patient Population

The electronic medical record database of the Department of General Surgery at the Second Affiliated Hospital of Xi’an Jiaotong University was reviewed, and the PH patients who underwent SPD from August 2009 to March 2017 were included in this study. Two SPD types were used in our department: splenectomy plus simplified pericardial devascularization (SSPD) and splenectomy plus traditional pericardial devascularization (STPD). The surgical details were described in our previous publications.^2^ The inclusion criteria were as follows: (i) PH patients with a history of upper gastrointestinal bleeding; (ii) PH patients diagnosed with esophagogastric varices and hypersplenism through clinical symptoms combined with laboratory, digestive endoscopy, or image examinations; (iii) PH patients classified as grade A or B in the Child–Pugh grading criteria or who were reduced to Child–Pugh A or B after liver preservation therapy to attain surgical indications; and (iv) patients who could tolerate general anesthesia and had no surgical contraindications. The exclusion criteria were as follows: (i) patients with acute heart failure, shock, or other vital organ diseases; (ii) patients in the acute hemorrhagic period with unstable vital signs; and (iii) patients with poor heart, lung, liver, or kidney functions. The patient characteristics, laboratory information, and perioperative characteristics were recorded from the electronic medical records. The study was reviewed and approved by the Ethics Committee of the Second Affiliated Hospital of Xi’an Jiaotong University (no.: 2018028). All procedures were conducted in accordance with the Declaration of Helsinki of the World Medical Association. The need to obtain written informed consent from the patients was waived due to the retrospective and anonymous nature of this study. All data were used only for statistical analysis in this study.

### Comprehensive Complication Index and Clavien–Dindo Classification for Evaluation of Morbidity

Complications that occurred within 30 days after the operation were considered surgical complications, and the severity of all complications was graded using the CDC.^4^ The CCI was the severity-weighted sum of all postoperative complications (available at http://www.assessurgery.com). The CDC grade was ascertained by the most serious complications for each patient. The range of the CCI is from 0 (no complications) to 100 (death). A CCI score greater than 26.2 (CCI ≥26.2) was considered a high CCI score based on prior studies.^8^

### Comprehensive Complication Index-Based Cumulative Sum Control Chart

Details of the CUSUM control technique were provided in previous publications.^11,13^ In this study, the CCI of the individual operation type from 2011 to 2017 was tested in the CUSUM chart using the average CCI from August 2009 to December 2010 as the target value. To compare surgical outcomes between the 2 operation types, the combined CUSUM chart from 2011 to 2017 was presented using the average overall CCI from August 2009 to December 2010 as the target value. Finally, overall monitoring of surgical outcomes in our department from 2011 to 2017 was depicted with a target value of the average overall CCI from August 2009 to December 2010.

### Statistical Analysis

Continuous variables are presented as the mean ± SD. Categorical variables are expressed as frequencies and percentages. Spearman’s correlation analysis was performed to test and compare the CCI and CDC grading systems. The correlation coefficient score of the CCI for a postoperative hospital stay (PHS) was compared to the CDC score. Univariate and multivariate logistic regression analyses were performed to identify risk factors for a high CCI score and a longer PHS. From the univariate analysis, factors with *P* < .1 were included in the multivariate analysis. The Statistical Package for the Social Sciences version 21.0 (IBM Corp.; Armonk, NY, USA) was used for all analyses. All *P* values are 2 sided, and *P* < .05 are considered significant.

## RESULTS

### Patient Demographics and Characteristics

A total of 659 patients were included. The baseline characteristics are displayed in Table 1. The mean age of these patients was 48.79 ± 10.95 years, and 339 (51.44%) were male. All patients in the study cohort had a history of cirrhosis. A total of 447 (67.83%) cases had the hepatitis B virus and 79 (11.99%) cases had hepatitis C. A Child–Pugh liver function grade of A was found in 323 (49.01%) patients, Child–Pugh B in 307 (46.59%) patients, and Child–Pugh C in 29 (4.40%) patients. On admission, 66 (10.02%) patients had mild esophageal varices, 217 (32.93%) had moderate esophageal varices, and 376 (57.06%) had severe esophageal varices. A total of 189 (28.68%) patients had portal hypertension gastropathy (PHG). A total of 28 (4.25%) patients had a history of variceal ligation, 166 (25.19%) had a history of smoking, 131 (19.88%) had a history of alcohol abuse, and 326 (49.47%) had a history of variceal bleeding. In terms of underlying diseases, 41 (6.22%) patients had hypertension, 65 (9.86%) suffered diabetes mellitus, 114 (17.30%) had cholecystolithiasis, and 313 (47.50%) had ascites. The mean BMI of these patients was 22.17 ± 3.17 (kg/m^2^), and the mean model for end-stage liver disease (MELD) score at admission was 5.96 ± 0.40.

### Laboratory Data and Intraoperative and Postoperative Characteristics

The laboratory Data and the intraoperative and postoperative characteristics of the cases are shown in Table 2. In this study, 236 (35.81%) patients underwent SSPD, and the other patients underwent STPD. The mean PHS was 14.94 ± 5.53 days, and the mean total hospitalization expense (THE) was 32 579.14 ± 17 395.22 (yuan). The operative time, intraoperative blood loss, and time to first flatus were 132.02 ± 50.02 minutes, 694.89 ± 607.68 mL, and 4.32 ± 1.56 days, respectively. When the preoperative imaging results were combined with the intraoperative assessment, the spleen thickness, spleen diameter, and portal vein diameter were 6.01 ± 1.34 cm, 16.82 ± 2.93 cm, and 1.45 ± 1.09 cm, respectively. Ascites (9.56%) and pneumonia (6.37%) were the most common complications after SPD. Portal vein thrombosis and surgical site infections occurred in 18 (2.73%) and 11 (1.67%) patients, respectively. Two patients suffered hepatic encephalopathy. Four (0.61%) patients underwent reoperation due to intra-abdominal bleeding. Four in-hospital deaths were observed in this cohort due to severe postoperative complications or organ failure.

### Comprehensive Complication Index and Clavien–Dindo Classification in Portal Hypertension Patients

**Figure 1.**
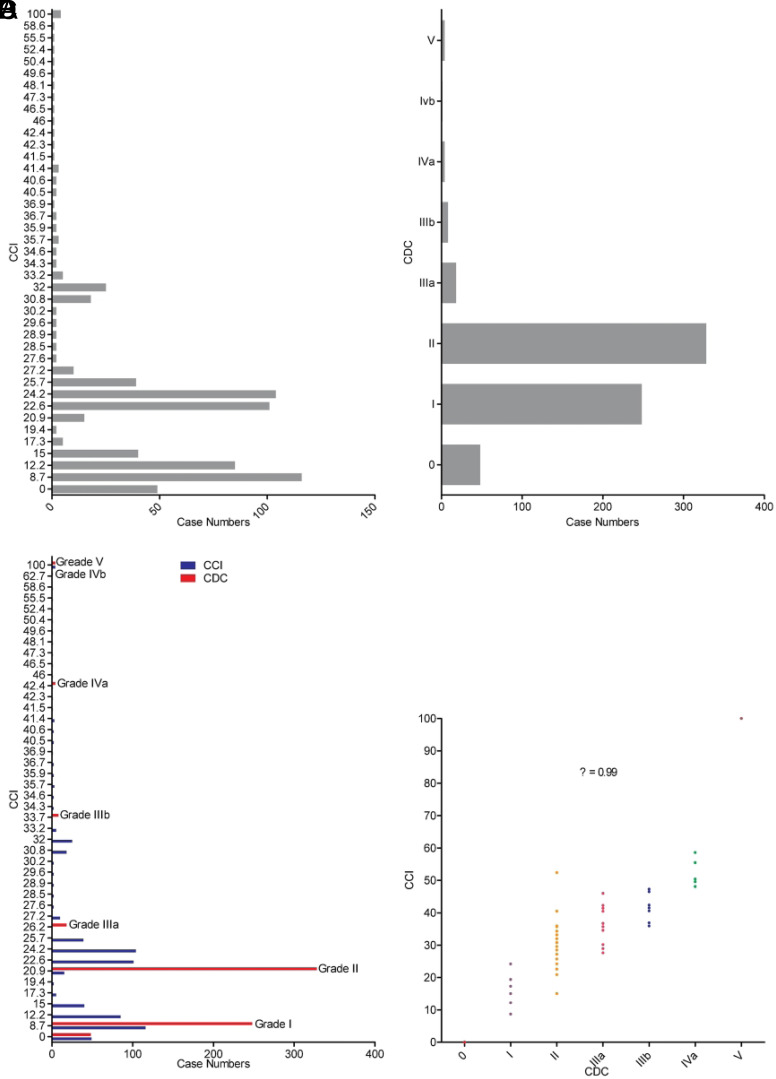
Comprehensive complication index (CCI) and Clavien–Dindo classification of portal hypertension patients. CCI, comprehensive complication index; CDC, Clavien–Dindo classification.

As shown in Table 3, the postoperative complications by CDC grade included 248 (40.59%) complications for grade I patients, 328 (53.68%) for grade II patients, 26 (4.26%) for grade III patients, and 9 (1.47%) for grade IV or higher (≥IV) patients. Among the 659 total patients, 611 (92.72%) developed complications, of whom 133 (21.77%) presented with a single complication and 478 (78.23%) presented with multiple complications. Complications following reoperation were integrated into the primary operation. The mean overall CCI was 19.22 ± 11.92. A total of 556 (84.37%) patients had a CCI score <26.2, and 103 (15.63%) patients had a CCI score >26.2. The distributions of complications according to the CCI and CDC are shown in Figure 1A and 1B. The CCI distribution was more discrete than that of the CDC because the CCI was a continuous scale and the CDC was an ordinal scale. When plotting the CCI and CDC for each individual patient on the same graph, the CCI varied incrementally for patients with the same CDC (Figure 1C). The same phenomenon was observed in the scatter diagram (Figure 1D). The correlation analysis found an extremely strong association between the CCI and CDC (correlation coefficient *ρ* = 0.99).

### Factors Associated with a High Comprehensive Complication Index

As shown in Table 4, we selected 38 clinical indicators for the univariate analyses. The results showed that the *P* values were all lower than .1 (<.10) for the Child–Pugh grade at admission, esophageal varices grade, variceal bleeding, hemoglobin, total bilirubin, direct bilirubin, albumin, serum creatinine, albumin–bilirubin grade, operative time, intraoperative blood loss, and spleen thickness. Then, we introduced these variables into the multivariate analysis and found that the Child–Pugh grade at admission, intraoperative blood loss and spleen thickness were independent risk factors for a high CCI score.

### Correlation of the Comprehensive Complication Index and Clavien–Dindo Classification with the Postoperative Hospital Stay and Total Hospitalization Expenses

The correlation analysis showed that both the CCI and CDC had positive correlations with the PHS and THE (Table 5). The CCI exhibited a stronger positive relationship with both the PHS (*ρ* = 0.2 vs. *ρ* = 0.15) and THE than the CDC (*ρ* = 0.23 vs. *ρ* = 0.19). The univariate and multivariate logistic regression analyses showed that the CCI and CDC were influenced by the PHS but not the THE. In the multivariate logistic regression model for the PHS, the *R*^2^ was 0.15 when we deleted the CDC and 0.14 when we deleted the CCI (Table 6).

### Cumulative Sum-Comprehensive Complication Index Charts Monitor Postoperative Complications

**Figure 2.**
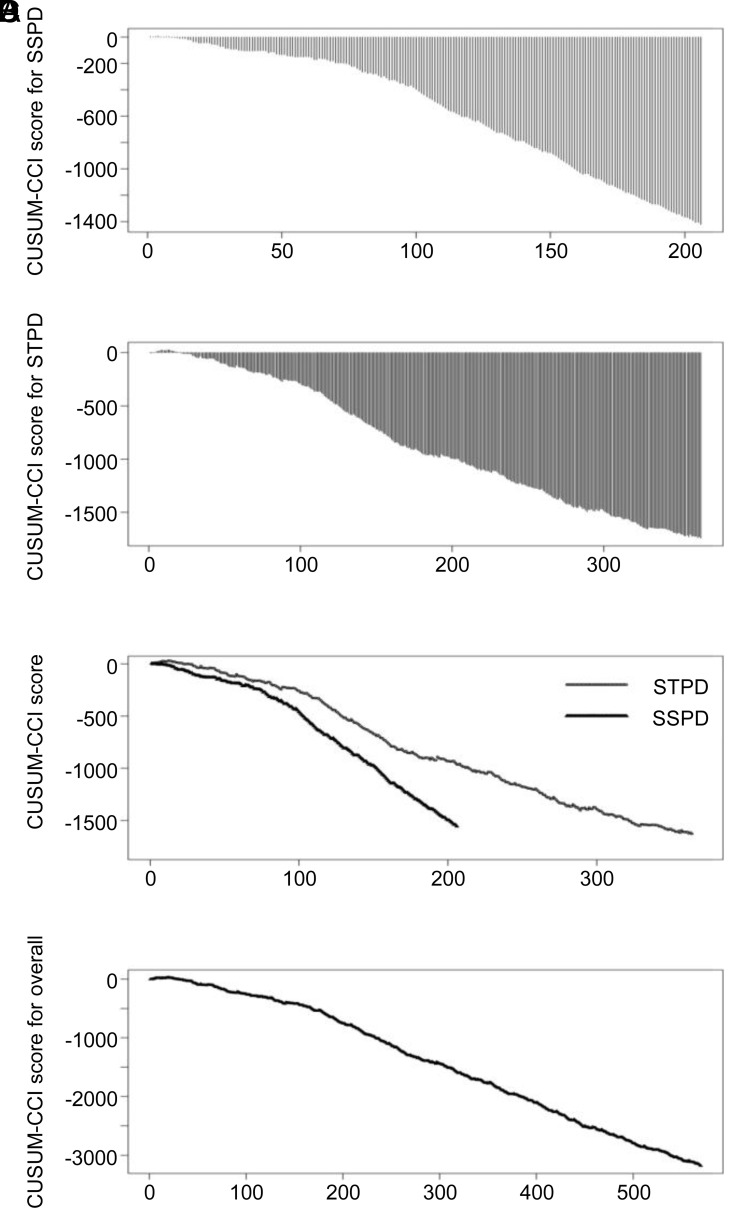
Application of CUSUM-CCI charts to monitor postoperative complications. CCI, comprehensive complication index; SSPD, splenectomy plus simplified pericardial devascularization; STPD, splenectomy plus traditional pericardial devascularization.

The complication outcomes of each operation type from 2011 to 2017 were monitored using the CUSUM-CCI chart. For SSPD, a target value of 23.85 was set for 208 cases. As shown in Figure 2A, the CUSUM score showed a gradual decrease until case 90 and then a sharp decrease from case 90 to case 208, resulting in a CUSUM-CCI score of –1421.43 in the last case and indicating improvement in the CCI. For STPD, a target value of 24.82 was applied for 366 cases. The CUSUM score showed a gradual decrease until the last case. The resulting CUSUM-CCI score for STPD was –1741.25, indicating improved complication outcomes compared to 2009 and 2010 (Figure 2B). To compare the surgical outcomes of the 2 operation types, the CUSUM-CCI chart was depicted for each operation type with a target value of 24.50. As shown in Figure 2C, SSPD exhibited a more notable decrease in the CUSUM score than STPD. The CUSUM chart for the overall CCI revealed a dynamic decrease in the time sequence, as shown in Figure 2D.

## DISCUSSION

The CCI is a novel method to evaluate postoperative complications that considers not only the numbers but also the severity of complications and is represented as a numeric scalev.^11^ Research has shown that numeric scales are the first choice to describe surgical complications.^14^ The traditional CDC system has many defects. This system uses only the most serious complications and ignores the collateral complications and the numbers of complications.^4^ In this study, 78.23% of patients had 2 or more complications, which were not captured by the CDC. Moreover, more severe complications are often accompanied by additional minor complications. However, the CDC does not capture the overall burden of complications in these cases and thus produces an insufficient report compared to that of the CCI. This study demonstrates the superiority of the CCI over the conventional CDC system in patients who have undergone SPD for PH. Slaman et al^15^ and Kim et al^11^ studied esophagectomy and radical gastrectomy, respectively, and obtained similar conclusions.

At present, reports of complications based on the CCI system are limited. In recent prospective studies, CCI values for esophagectomy of 25.74 (8.66–43.01),^16^ hepatectomy of 28 (range, 0-100),^17^ liver transplantation (median 37.1),^18^ and cytoreduction and hyperthermic intraperitoneal chemotherapy (25.3 ± 24.1)^19^ have been reported. According to these previous reports and our current report regarding the use of the CCI for SPD (19.22 ± 11.92), the CCI appears to be a good method to evaluate postoperative complications. To the best of our knowledge, this study is the first to report the postoperative complications of PH patients undergoing SPD using the CCI system. The CCI score ranges from 0 (no complications) to 100 (death).^20^ Patients with a CCI score ≥26.2 are considered to have a severe postoperative condition.^21^ Here, we found that >15.63% of cirrhotic patients had a postoperative CCI score >26.2. Ascites occurred in 9.56% and pneumonia in 6.37% of the patients. Portal vein thrombosis and surgical site infections occurred in 18 (2.73%) and 11 (1.67%) patients, respectively. Two patients suffered hepatic encephalopathy. Four (0.61%) patients underwent reoperation due to intra-abdominal bleeding. Four in-hospital deaths were observed in this cohort due to severe postoperative complications or organ failure. Thus, severe postoperative conditions are common in PH patients undergoing SPD.

Identification of the risk factors for a high CCI score is critical for the prediction and prevention of severe complications.^22^ In this study, we found that a high Child–Pugh grade at admission was associated with a high CCI score after SPD. The Child–Pugh classification is a commonly used grading standard for liver reserve function, liver parenchymal damage, and prognostic predictions in cirrhotic patients. Patients with high Child–Pugh grades are more likely to experience postoperative liver dysfunction, which potentially increases the surgical risk.^23^ This study is the first to show the association of this measure with a high CCI score after SPD in PH patients. Spleen thickness was another risk factor for a high CCI score. Spleen thickness was associated with the severity of cirrhosis.^24^ Patients with thicker spleens are more likely to experience postoperative liver dysfunction and coagulation abnormalities due to the cirrhosis severity, resulting in more complications and a high CCI. Thus, other treatment options should be considered for PH patients with high Child–Pugh classifications and thicker spleens. Intraoperative blood loss was another risk factor for a high CCI score. Intraoperative blood loss is a potential complication of surgery. Studies have shown that the risk of serious morbidity and mortality from surgery increases with the increase in intraoperative blood loss.^25^ Severe intraoperative blood loss can lead to postoperative anemia and even shock, especially in PH patients with poor liver function. Therefore, intraoperative blood loss should be reduced as much as possible to reduce complications.

A PHS and THE are considered surrogate markers that reflect the clinical outcome.^11^ In this study, the correlation analysis showed that both the CCI and CDC had positive correlations with the PHS and THE, although the CCI exhibited a stronger positive relationship with both the PHS and THE than the CDC. The univariate and multivariate logistic regression analyses showed that the CCI and CDC were both influenced by the PHS but not by the THE, which might have been due to the different types of medical insurance used by individual patients. The *R*^2^ of the multivariate analysis was 0.15 when we deleted the CDC and 0.14 when we deleted the CCI, suggesting that the CCI had a greater share of the influence on the PHS than the CDC.^26^

Because the CCI system is a continuous variable, it can be mathematically integrated to form the CUSUM-CCI control chart to monitor and compare the CCIs of different operation types and to monitor overall surgical outcomes. This method can continuously update complication data and be used to instantly observe the postoperative effect and finally to provide feedback for different surgical types to reassess operation procedures. In this study, the CUSUM score showed improvement in the CCI for both SSPD and STPD. In the comparison model, SSPD exhibited a more notable decrease in the CUSUM score than STPD, indicating that SSPD was superior to STPD in terms of the CCI score and was safe, effective, simple, and easy to perform.^2^ The CUSUM chart for the overall CCI revealed a dynamic decrease in the time sequence, indicating that the total postoperative complications were decreasing.

Nevertheless, our study still has some limitations. First, this study was a retrospective study, and thus we cannot exclude potential selection bias and residual confounding due to unknown covariates that may have affected our results.^27^ Second, our study was performed in a single hospital. Thus, potential information on readmission to other hospitals was not included. Third, because the follow-up time in our study was only 1 month, we could not report the longer-term postoperative outcomes of PH patients. Despite the above limitations, we believe that our study contributes to complication evaluation and monitoring of SPD for PH patients.

## CONCLUSION

In conclusion, the CCI is an effective tool to assess the overall burden of postoperative complications for PH patients undergoing SPD. The Child–Pugh classification at admission, spleen thickness, and intraoperative blood loss were associated with a high CCI score after SPD. Thus, an elevated Child–Pugh grade at admission and a thick spleen indicate that other treatment options should be considered for PH. Surgeons should make every effort to optimize surgical decision-making and minimize intraoperative blood loss. This study also found that the CCI exhibited a stronger correlation with complications and better predicted the PHS and THE than the conventional CDC. In addition, the CUSUM-CCI control model can be used to continuously monitor and compare the surgical quality among different operation types. Further, large-scale prospective studies are needed to confirm our findings.

## Figures and Tables

**Table 1.** Patient Demographics and Characteristics (n = 659)

Variable	Mean ± SD or n (%)
Age, years	48.79 ± 10.95
Sex, male	339 (51.44%)
Etiology	
Hepatitis B	447 (67.83%)
Hepatitis C	79 (11.99%)
Others	133 (20.18%)
Child–Pugh grade at admission	
A	323 (49.01%)
B	307 (46.59%)
C	29 (4.40%)
Esophageal varices grade	
Mild	66 (10.02%)
Moderate	217 (32.93%)
Severe	376 (57.06%)
Charlson score	
0	356 (54.02%)
1	186 (28.22%)
2	78 (11.84%)
≥3	39 (5.92%)
Coexisting conditions	
PHG	189 (28.68%)
Variceal ligation	28 (4.25%)
Smoking	166 (25.19%)
Drinking	131 (19.88%)
Variceal bleeding	326 (49.47%)
Hypertension	41 (6.22%)
Diabetes	65 (9.86%)
Cholecystolithiasis	114 (17.30%)
Ascites	313 (47.50%)
BMI	22.17 ± 3.17
MELD score at admission	5.96 ± 0.40

BMI, body mass index; MELD, model for end-stage liver disease; PHG, portal hypertension gastropathy.

**Table 2.** Laboratory Data and Perioperative Characteristics

Variable	Mean ± SD or n (%)
WBC (10^9^/L)	2.71 ± 1.96
Hb (g/L)	94.22 ± 25.65
Platelet (10^9^/L)	47.4 ± 25.75
PT (s)	13.87 ± 1.91
INR	1.2 ± 0.50
TBIL (µmol/L)	27.52 ± 16.62
DBIL (µmol/L)	11.32 ± 6.65
ALT (IU/L)	38.91 ± 42.09
AST (IU/L)	46.54 ± 41.81
ALB (g/L)	36.55 ± 5.59
GLB (g/L)	28.64 ± 5.99
Scr (µmol/L)	64.79 ± 18.76
Cys C (mg/L)	1.14 ± 0.33
ALBI	
1	143 (21.70%)
2	474 (71.93%)
3	42 (6.37%)
Postoperative hospital stay (days)	14.94 ± 5.53
Total hospitalization expense (yuan)	32 579.14 ± 17 395.22
Operation time (minutes)	132.02 ± 50.02
Intraoperative blood loss (mL)	694.89 ± 607.68
Time to first flatus (days)	4.32 ± 1.56
Spleen thickness (cm)	6.01 ± 1.34
Spleen diameter (cm)	16.82 ± 2.93
Portal vein diameter (cm)	1.45 ± 1.09
Operation types (SSPD)	236 (35.81%)
Postoperative complications	
Ascites	63 (9.56%)
Pneumonia	42 (6.37%)
Portal vein thrombosis	18 (2.73%)
Surgical site infection	11 (1.67%)
Hepatic encephalopathy	2 (0.30%)

ALB, albumin; ALBI, albumin–bilirubin grade; ALT, alanine transaminase; AST, aspartate transaminase; Cys C, cystatin C; DBIL, direct bilirubin; GLB, globulin; Hb, hemoglobin; INR, international normalized ratio; PT, prothrombin time; Scr, serum creatinine; SSPD, splenectomy plus simplified pericardial devascularization; TBIL, total bilirubin; WBC, white blood cell.

**Table 3.** Complications According to the CDC and CCI

Variable	Mean ± SD or n (%)
Patients with complications	611 (92.72%)
Clavien–Dindo classification	
I	248 (40.59%)
II	328 (53.68%)
IIIa	18 (2.95%)
IIIb	8 (1.31%)
IVa	4 (0.65%)
Ivb	1 (0.16%)
V	4 (0.65%)
Number of complications	
1	133 (21.77%)
2	190 (31.10%)
3	172 (28.15%)
4	81 (13.26%)
5	28 (4.58%)
6	6 (0.98%)
7	1 (0.16%)
Overall CCI	19.22 ± 11.92
CCI <26.2	556 (84.37%)
CCI ≥26.2	103 (15.63%)

CCI, comprehensive complication index; CDC, Clavien–Dindo classification.

**Table 4.** Univariate and Multivariate Analyses of the Factors Associated with a High CCI

Variable	CCI <26.2 (n = 556)	CCI ≥26.2 (n = 103)	Univariate analysis (*P*)	OR (95% CI)	Multivariate analysis (*P*)
Age (years)	48.69 ± 10.82	49.33 ± 11.67	.59		
Sex (male)	281 (50.54%)	58 (56.31%)	.28		
Etiology			.79		
Hepatitis B	380 (68.35%)	67 (65.05%)			
Hepatitis C	65 (11.69%)	14 (13.59%)			
Others	111 (19.96%)	22 (21.36%)			
Child–Pugh grade at admission			**.01**	(-)	**.04 **
A	286 (51.44%)	37 (35.92%)		—	—
B	244 (43.88%)	63 (61.17%)		1.49 (0.86-2.58)	.15
C	26 (4.68%)	3 (2.91%)		0.35 (0.08-1.59)	.17
Esophageal varices grade			**.07**	—	.19
Mild	55 (9.89%)	11 (10.68%)		—	—
Moderate	193 (34.71%)	24 (23.30%)		0.51 (0.22-1.16)	.11
Severe	308 (55.40%)	68 (66.02%)		0.77 (0.36-1.65)	.51
PHG	158 (28.42%)	31 (30.10%)	.73		
Charlson score			.73		
0	300 (53.96%)	56 (54.37%)			
1	155 (27.88%)	31 (30.10%)			
2	69 (12.41%)	9 (8.74%)			
≥3	32 (5.76%)	7 (6.80%)			
Variceal ligation	24 (4.32%)	4 (3.88%)	.84		
Hypertension	35 (6.29%)	6 (5.83%)	.86		
Diabetes	54 (9.71%)	11 (10.68%)	\.76		
Cholecystolithiasis	95 (17.09%)	19 (18.45%)	.74		
Smoking	142 (25.54%)	24 (23.30%)	.63		
Drinking	113 (20.32%)	18 (17.48%)	.51		
Variceal bleeding	267 (48.02%)	59 (57.28%)	**.08**	1.24 (0.73-2.09)	.42
Ascites	269 (48.38%)	44 (42.72%)	.29		
BMI	22.14 ± 3.19	22.30 ± 3.05	.99		
MELD score at admission	5.95 ± 0.40	6.02 ± 0.43	.14		
Portal vein diameter (cm)	1.46 ± 1.16	1.39 ± 0.15	.33		
WBC (10^9^/L)	2.71 ± 1.97	2.77 ± 1.89	.77		
Hb (g/L)	95.33 ± 25.99	88.08 ± 22.88	**.01**	0.99 (0.98-1.003)	.17
Platelet (10^9^/L)	47.38 ± 25.49	47.55 ± 27.25	.95		
PT (s)	13.87 ± 1.88	13.87 ± 2.07	.99		
INR	1.20 ± 0.54	1.18 ± 0.18	.67		
TBIL (µmol/L)	26.93 ± 15.74	30.74 ± 20.58	**.03 **	1.02 (0.99-1.04)	.13
DBIL (µmol/L)	11.12 ± 6.24	12.42 ± 8.51	**.06 **	0.99 (0.94-1.05)	.89
ALT (IU/L)	38.26 ± 32.20	42.46 ± 76.18	.36		
AST (IU/L)	46.04 ± 34.22	49.28 ± 70.14	.48		
ALB (g/L)	36.82 ± 5.58	35.08 ± 5.47	**.00 **	0.99 (0.92-1.07)	.85
GLB (g/L)	28.77 ± 5.87	27.94 ± 6.62	.20		
Scr (µmol/L)	64.23 ± 18.58	67.82 ± 19.48	**.08 **	1.01 (0.99-1.01)	.23
Cys C (mg/L)	1.13 ± 0.32	1.18 ± 0.39	.22		
ALBI			**.04**		.83
1	130 (23.38%)	13 (12.62%)		—	—
2	393 (70.68%)	81 (78.64%)		0.95 (0.38-2.39)	.91
3	33 (5.94%)	9 (8.74%)		0.67 (0.12-3.68)	.64
Operative time (minutes)	129.71 ± 46.12	147.13 ± 69.12	**.00 **	0.99 (0.99-1.01)	.79
Intraoperative blood loss (mL)	658.28 ± 549.86	934.82 ± 868.22	**.00 **	1.01 (1.0-1.10)	**.01 **
Time to first flatus (days)	4.32 ± 1.57	4.33 ± 1.46	.98		
Spleen thickness (cm)	5.98 ± 1.34	6.19 ± 1.29	**.02 **	1.39 (1.02-1.91)	**.04 **
Spleen diameter (cm)	16.91 ± 2.61	16.21 ± 4.63	.63		
Operation types (SSPD)	204 (36.69%)	32 (31.07%)	.27		

ALB, albumin; ALBI, albumin–bilirubin grade; ALT, alanine transaminase; AST, aspartate transaminase; BMI, body mass index; CCI, comprehensive complication index; Cys C, cystatin C; DBIL, direct bilirubin; GLB, globulin; Hb, hemoglobin; INR, international normalized ratio; MELD, model for end-stage liver disease; OR, odds ratio; PHG, portal hypertension gastropathy; PT, prothrombin time; Scr, serum creatinine; SSPD, splenectomy plus simplified pericardial devascularization; TBIL, total bilirubin; WBC, white blood cell.

**Table 5.** Correlation Coefficient Scores of the CCI and CDC with Postoperative Hospital Stay and Total Hospitalization Expense

Variables	Correlation index (ρ)	*P*
Postoperative hospital stay		
CCI	0.20	*P* < .001
CDC	0.15	*P* < .001
Total hospitalization expense		
CCI	0.23	*P* < .001
CDC	0.19	*P* < .001

CCI, comprehensive complication index; CDC, Clavien–Dindo classification.

**Table 6.** Multivariate Analysis of the Factors Affecting PHS

Variables	*B*	Standard error	OR (95% CI)	*P*	*R* ^2^
CCI and postoperative hospital stay					
CCI	0.04	0.01	1.04 (1.02-1.06)	<.05	0.15
PHG	0.33	0.21	1.38 (0.92-2.09)	.12	
Ascites	0.27	0.20	1.31 (0.89-1.93)	.16	
Esophageal varices grade				.69	
Esophageal varices grade (1)	0.11	0.37	1.12 (0.55-2.29)	.76	
Esophageal varices grade (2)	0.25	0.35	1.28 (0.65-2.55)	.48	
Diabetes	0.72	0.31	2.05 (1.11-3.80)	<.05	
Operative time (minutes)	0.02	0.003	1.01 (1.01-1.02)	<.05	
Intraoperative blood loss (mL)	–0.0004	0.0003	0.99 (0.99-1.01)	.15	
Scr (µmol/L)	0.01	0.01	1.01 (0.99-1.02)	.26	
Intraoperative transfusion (mL)	0.0004	0.0003	1.01 (0.99-1.01)	.19	
Constant	–3.64	0.60	0.03	<.05	
CDC and postoperative hospital stay					
CDC				<.05	0.14
I	0.66	0.43	1.93 (0.83-4.47)	.12	
II	0.85	0.43	2.34 (1.01-5.46)	<.05	
IIIa	2.12	0.71	8.31 (2.08-33.13)	<.05	
IIIb	2.24	0.98	9.36 (1.38-63.57)	<.05	
>IVa	0.33	1.13	1.39 (0.15-12.64)	.77	
PHG	0.32	0.21	1.38 (0.91)	.13	
Ascites	0.28	0.20	1.32 (0.90-1.95)	.16	
Esophageal varices grade				.58	
Esophageal varices grade (1)	0.11	0.36	1.11 (0.54-2.27)	.77	
Esophageal varices grade (2)	0.28	0.35	1.33 (0.67-2.63)	.42	
Diabetes	0.73	0.31	2.07 (1.12-3.82)	<.05	
Operative time (minutes)	0.01	0.003	1.01 (1.01-1.02)	<.05	
Intraoperative blood loss (mL)	–0.0003	0.0003	0.99 (0.99-1.01)	.29	
Scr (µmol/L)	0.01	0.006	1.01 (0.99-1.02)	.16	
Intraoperative transfusion (mL)	0.0004	0.0003	1.01 (0.99-1.01)	.20	
Constant	–3.83	0.70	0.02	.05	

CCI, comprehensive complication index; OR, odds ratio; PHG, portal hypertension gastropathy, PHS, postoperative hospital stay; Scr, serum creatinine.
